# Optimization of immunomagnetic separation for cord blood-derived hematopoietic stem cells

**DOI:** 10.1186/1471-2121-7-30

**Published:** 2006-08-01

**Authors:** Tuija Kekarainen, Sirkka Mannelin, Jarmo Laine, Taina Jaatinen

**Affiliations:** 1 Finnish Red Cross Blood Service, Helsinki, Finland; 2 Centre de Recerca en Sanitat Animal (CReSA), Universitat Autònoma de Barcelona, Barcelona, Spain

## Abstract

**Background:**

There is a growing interest in cord blood as a source of primitive stem cells with the capacity for multilineage differentiation. Pure cell fractions are needed for the characterization and *in vitro *expansion of stem cells as well as for their use in preclinical research. However, enrichment of stem cells is challenging due to the lack of stem cell-specific markers and gentle protocols for the isolation of highly pure stem cell fractions. Protocols developed for the enrichment of peripheral blood-derived stem cells have been found to be suboptimal for cord blood.

**Results:**

In this study, immunomagnetic cell sorting protocols to purify CD34+, CD133+ and Lin- cells from fresh and cryopreserved cord blood were optimized. Reproducible purities of up to 97% were reached. The selected cells were highly viable having substantial colony-forming potential.

**Conclusion:**

The optimized protocols enable rapid enrichment of highly pure hematopoietic stem cells from both fresh and cryopreserved cord blood.

## Background

Hematopoietic stem cells (HSC), with their unique self-renewal and differentiation capacity, offer great potential for the treatment of hematological disorders, immunodeficiency and inborn errors of metabolism [1,2]. HSCs can be collected from mobilized peripheral blood (PB), bone marrow (BM) and cord blood (CB). Lately, CB has been increasingly utilized because it is readily available, HLA mismatches are better tolerated and there is a decreased risk of graft-versus-host disease when using CB-derived HSCs when compared to the other sources [3]. Even though the cell content of CB is limited, it has a higher frequency of progenitor cells compared to PB or BM [3-5]. CB-derived CD34+ cells have also been shown to proliferate more rapidly than their counterparts from BM [6], and CB-derived HSCs possess increased engraftment potential when compared to cells from PB or BM [7,8]. In addition, recent studies suggest that CB is a source of non-hematopoietic stem or progenitor cells, such as mesenchymal and endothelial precursors [9,10].

Enrichment of HSCs is based on the expression of certain surface antigens or on the lack of expression of lineage-specific antigens. The most commonly used surface marker for HSC selection is the transmembrane glycoprotein CD34. CD34 is also used to quantify the stem cell content of CB units banked for clinical use [11]. Most, if not all, CD34+ cells express the CD133 glycoprotein on their surface. CD133 appears to be expressed on more primitive cells and CD133+ cell grafts have been tested in stem cell transplantation [12-14]. Alternative, but currently poorly characterized Lin- progenitor cells lack lineage-specific markers [15,16]. Fluorescence-activated cell sorting and immunomagnetic selection systems utilize antibodies against these cell surface antigens to enrich HSCs. However, the major challenge has been the difficulty to produce highly pure HSC fractions from CB with good recovery. Furthermore, the handling of CB is challenging due to the relatively high content of thrombocytes and nucleated erythroid precursors which have a negative impact on the mononuclear cell isolation. For these reasons, standardized protocols for PB sample handling and cell separation do not work well for CB. Only few studies have investigated the efficiency of the immunomagnetic selection method used to isolate CD34+ cells from CB. Belvedere et al. compared the results from 49 selections and reported mean CD34+ cell purities of 41% and 85% after first and second passage through the separation column, respectively [17]. Melnik et al. report an average purity of 60% for CB-derived CD34+ cells from 10 separations [18].

In this study, three different protocols were optimized to enrich CD34+, CD133+ and Lin- HSCs with over 90% purity from both fresh and cryopreserved CB. Cryopreserved CB cells have been considered to be especially challenging in selection procedures because of cell aggregation caused by cell damage during thawing. The used protocols were based on positive selection of cells expressing CD34 and CD133, or on depletion of cells expressing lineage-specific markers. The magnetic cell sorting system MACS was used due to its gentleness and time-effectiveness. Further, the clonogenic capacity of selected HSC populations was determined using the colony-forming unit (CFU) assay.

## Results and discussion

### Handling of CB cells

The isolation of pure mononuclear cell (MNC) fractions from CB, and subpopulations thereof, brings about a special challenge. This appears to be due to the large number of thrombocytes and erythroid progenitors in CB. In the Ficoll-Paque density gradient, all erythroid cells do not necessarily sediment to the bottom layer, but are retained in the interphase of plasma and Ficoll-Paque. The erythroid cells remaining in the interphase are nucleated progenitors that may hamper the subsequent immunomagnetic selection of HSC populations. Treatment with ammonium chloride or diethylene glycol may be used to deplete red blood cells, but depletion was not performed in this study as the nucleated erythroid progenitors are not easily lyzed and purities up to 97% were reached without any additional treatment. The unusually slow sedimentation of erythroid cells is not seen when working with PB.

When handling cryopreserved CB cells, aggregation was observed. Aggregation was reduced by replacing ethylenediamine tetraacetic acid (2 mM EDTA, Merck, Darmstadt, Germany) with anticoagulant citrate dextrose solution, formula A (0.6% ACD/A, Baxter Healthcare, Lessines, Belgium) in the sample buffer. In some cases aggregation was so substantial that the cells needed to be resuspended in DNaseI containing buffer. DNaseI digests the DNA released from dead cells and prevents aggregation. The DNase treatment did not affect the viability or colony-forming potential of selected CB cells. No cell aggregation was seen when handling fresh CB cells.

### MNC fraction

**Figure 1:**
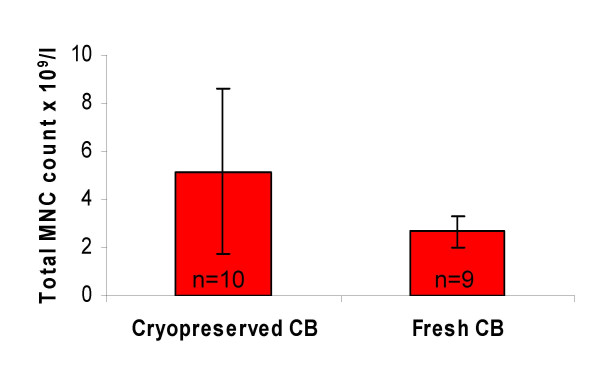
**Mean MNC count in cryopreserved and fresh CB**. The mean MNC concentration for cryopreserved and fresh CB was 5.14 × 10^9^/l (range 0.96–10.00, median 4.30, SD = 3.43) and 2.68 × 10^9^/l (range 1.24–3.62, median 2.77, SD = 0.64), respectively. The difference between cryopreserved and fresh CB was not statistically significant (P = 0.06). Bars show means and error bars show standard deviations. Abbreviations: MNC, mononuclear cells; CB, cord blood.

In cryopreserved CB, the mean MNC concentration was 5.14 × 10^9^/l (range 0.96–10.00, SD = 3.43) (Figure 1), and the mean platelet concentration was 8.30 × 10^9^/l (range 0.00–17.00, SD = 5.46). Cryopreserved CB contained a mean of 0.14 × 10^12^/l erythrocytes (range 0.02–0.67, SD = 0.20), and the mean hematocrit was 2%.

Fresh CB contained a mean of 2.68 × 10^9^/l MNCs (range 1.24–3.62, SD = 0.64) (Figure 1). The difference in MNC concentration between cryopreserved and fresh samples was not statistically significant (P = 0.06). The remarkably high disparity in the standard deviation of MNC concentration between cryopreserved and fresh CB may be due to the processing and freezing of cells performed to bank the CB units [19]. The mean concentration was 205.89 × 10^9^/l (range 84.00–505.00, SD = 130.58) for platelets and 0.12 × 10^12^/l (range 0.03–0.50, SD = 0.14) for erythrocytes. The mean hematocrit was 1%.

### Immunomagnetic separation of HSC populations

**Figure 2:**
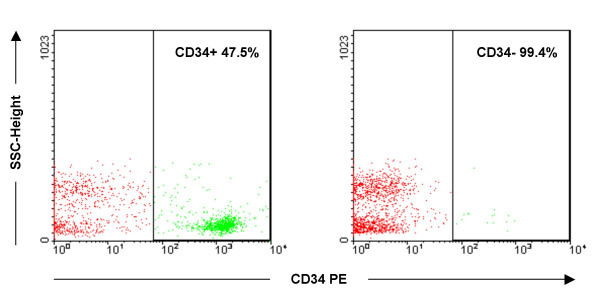
**Purity assessment of the CD34+ cell fraction by flow cytometry**. The initial purity of CD34+ cells after separation through single column was 47.5%. The CD34- fraction was 99.4% pure. CD34+ and CD34- cell populations were defined by first gating on forward and side scatter properties excluding platelets and debris. Subsequent gates were set to exclude >99% of control cells labeled with isotype-specific antibody. Percentages indicating the purity of isolated cell fractions are shown for both plots. Abbreviations: SSC, side scatter; PE, phycoerythrin.

**Figure 3:**
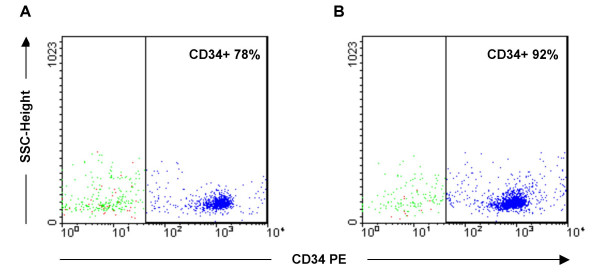
**Purity assessment of CD34+ cell fraction after one or two column separations**. A) The CD34+ cell fraction was 78% pure after the first column separation. B) A 92% pure CD34+ cell faction was obtained by an additional labeling step in connection with a second column separation. CD34+ cell populations were defined by first gating on forward and side scatter properties excluding platelets and debris. Subsequent gates were set to exclude >99% of control cells labeled with isotype-specific antibody. Percentages indicating the purity of isolated cell fractions are shown for both plots. Abbreviations: SSC, side scatter; PE, phycoerythrin.

When using the Direct CD34 Progenitor Cell Isolation Kit with single column separation and the labeling protocol recommended by the manufacturer (Miltenyi Biotec, Bergisch Gladbach, Germany), a purity of less than 50% was reached for the CD34+ cells (Figure 2). To obtain highly pure CD34+ cells, the immunomagnetic selection method was optimized. Several washing steps (3–10) were tested for single column separation. A purity of 80% was achieved with extensive washing, but the yield was poor (less than 50% of the expected yield). Two successive column separations resulted in 77% purity, but a great number of CD34+ cells were still lost during the process indicating a further need to optimize the protocol. An additional labeling step between the two column separations increased the purity to >90% (results for a representative sample shown in figure 3) and resulted in an acceptable yield as well. The optimized two-column method with additional labeling proved reliable and was applied to the separation of both CD34+ and CD133+ cells. The average yield of CD34+ and CD133+ cells from one cord blood unit was 10^6 ^and 10^5^, respectively. The purity of positively selected CD34+/CD133+ cells was reproducibly over 90% and their negative counterparts were nearly 100% pure.

**Figure 4:**
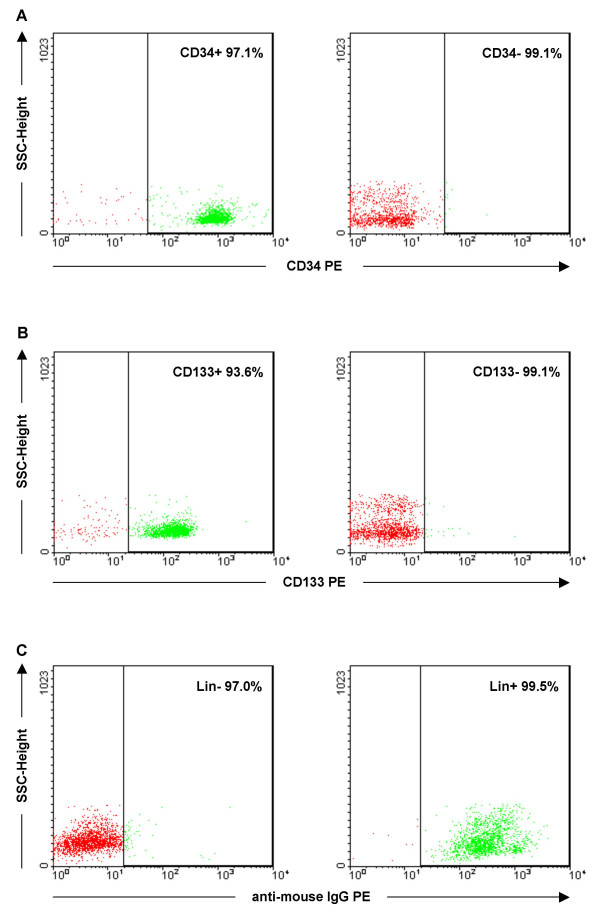
**Purity assessment of CD34+/-, CD133+/- and Lin-/+ cell fractions**. A) Purities for CD34+ and CD34- cell factions were 97.1% and 99.1%, respectively. B) Purities for CD133+ and CD133- fractions were 93.6% and 99.1%, respectively. C) Purities for Lin- and Lin+ cell factions were 97.0% and 99.5%, respectively. CD34+/-, CD133+/- cell populations were defined by first gating on forward and side scatter properties excluding platelets and debris. Subsequent gates were set to exclude >99% of control cells labeled with isotype-specific antibody. Percentages indicating the purity of isolated cell fractions are shown for both plots. Abbreviations: SSC, side scatter; IgG, immunoglobulin; PE, phycoerythrin.

Generally, the recovery of CD34+ and CD133+ cells was 0.86% (range 0.56–1.45, SD = 0.36) and 0.21% (range 0.04–0.41, SD = 0.12), respectively. The recovery of CD34+ cells was higher from fresh CB (0.97%) when compared to cryopreserved CB (0.78%), although the difference was not statistically significant (P= 0.54). The results are consistent with the study by Almici et al. showing no significant difference in yield or in purity for fresh CB CD34+ cells in comparison to crypreserved cells [20]. This was the case with CD133+ cells as well, the recovery being 0.29% for fresh CB and 0.12% for cryopreserved CB (P = 0.11). The purities were not affected by the initial percentage of HSC populations in CB. The results of the purity assessment for representative samples of CD34+/-, CD133+/- and Lin-/+ cells are shown in figure 4.

The magnetic sorting of Lin- cells was optimized to find the optimum concentrations for antibodies and magnetic colloids. Lin- cells were separated from the MNC fraction with magnetic cell sorting and the purity of the enriched cell fraction was analyzed by flow cytometry. The average yield of Lin- cells from one cord blood unit was 10^6^. The overall recovery of Lin- cells was 0.29% (range 0.13–0.70, SD = 0.21), being higher in fresh CB samples (0.43%) than in cryopreserved CB samples (0.16%). The difference between fresh and cryopreserved samples was not statistically significant (P = 0.19). The mean purity of Lin- cell population was 90% (range 82–98%, SD = 5.00) and it did not differ between fresh and cryopreserved CB units. To assess the effect of antibody concentration on the selection of Lin- cells, varying amounts of the antibody cocktail (50–100 μl/ml) and magnetic colloids (30–60 μl/ml) were tested. The concentration of antibodies and magnetic colloids did not affect the purity of Lin- cells based on flow cytometric analysis.

**Figure 5:**
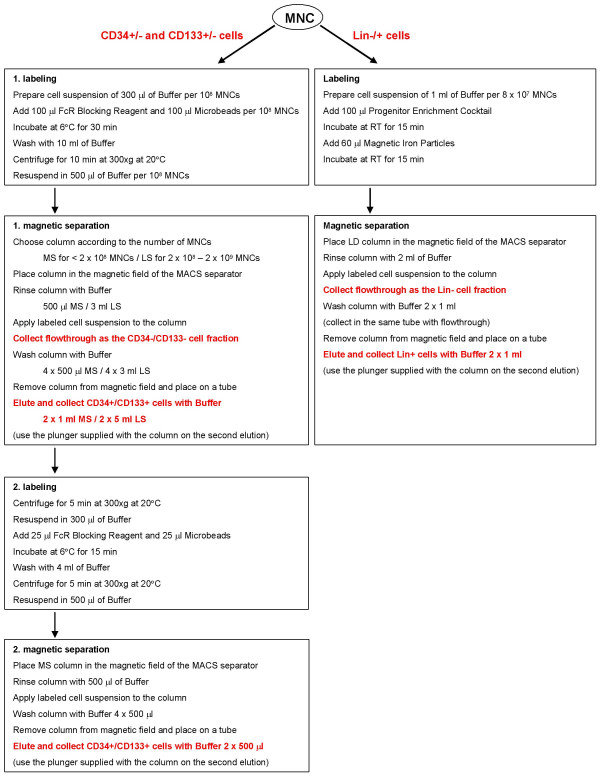
**A chart of the optimized protocols to isolate CD34+/-, CD133+/- and Lin-/+ cells from cord blood**. Isolation of CD34/- and CD133+/- cells was performed using Direct CD34 Progenitor Cell Isolation Kit (#130-046-702, Miltenyi Biotec) and CD133 Cell Isolation Kit (#130-050-801, Miltenyi Biotec), respectively. Lin-/+ cells were isolated using StemSep Human Progenitor Enrichment Kit (#14056, StemCell Technologies). For all magnetic separations, MACS columns and separators (Miltenyi Biotech) were used. Abbreviations: MNC, mononuclear cells; Buffer, PBS pH 7.2 supplemented with 0.5% bovine serum albumin and 2 mM EDTA or 0.6% ACD/A.

All the optimized protocols are described in detail in figure 5. The operation time for the selection of CD34+/- and CD133+/- cells using MiniMACS or MidiMACS is approximately 1.5 hours. The operation time for isolation of Lin-/+ cells is approximately 45 minutes. The selection can be made more effective with the AutoMACS system developed for high-speed automated cell sorting. The optimized protocols have been developed for enrichment of CB HSCs for research purposes. However, the enrichment of stem and progenitor cells is often necessary in clinical settings. The selected HSCs are increasingly used in transplantations and enrichment may be required for depletion of contaminating mature cells or tumor cells. Potentially, the methods described here could be applied to clinical-grade selection using the CliniMACS System that is CE-marked for clinical use in Europe.

With the optimized protocols, a purity of at least 90% was achieved for CD34+, CD133+ and Lin- cells. Viability was 99% for all the selected cell types. This demonstrates that the optimized protocols work well for HSC enrichment from both fresh and cryopreserved CB. Fresh CB was easier to handle and the recovery of HSCs was higher from fresh CB. Nonetheless, cryopreserved cord blood is almost exclusively used in clinical settings. Therefore, if one wishes to use selected HSCs, the cells should preferably be isolated on fresh cord blood and cryopreserved after the selection procedure to maximize the recovery of HSCs. HSCs, enriched by the protocols described here, have been used in gene expression studies with great reproducibility and consistency [21].

It has been suggested that the binding of an antibody to the surface of a HSC may influence cell proliferation and differentiation by activating intracellular signaling pathways [14]. An anti-CD34 antibody has been shown to induce tyrosine phosphorylation in BM-derived CD34+ cells [22]. However, Lin- cells are selected through negative depletion. Thus, neither antibody binding nor activation of signaling pathways is expected. Further studies on the effect of the interaction between HSCs and the antibodies used for their selection as well as the possible impact of this contact on HSC quality are awaited.

Low number of HSCs in cord blood is a limitation for its use. *Ex vivo *expansion of HSCs may be used to generate the clinically relevant cell numbers needed for adult patients. However, mature cells may develop during long-term culture and result in a need for reselection of progenitor cells. The optimized protocols can be applied for enrichment of stem and progenitor cells after *ex vivo *expansion.

### Colony forming unit assay

The CFU assay was used to measure the clonogenic capacity of CD34+, CD133+ and Lin- cells as well as MNCs. Total CFU (CFU-TOT) number was determined as the sum of granulocyte-erythroid-macrophage-megakaryocyte (CFU-GEMM), granulocyte-macrophage (CFU-GM), erythroid (CFU-E) and burst-forming erythroid (BFU-E) colonies. CFU-TOT counts were 84.5, 80.0, 57.3 and 0.5 per 1000 cells for CD34+, CD133+, Lin- and MNCs, respectively. CD34- and CD133- cell populations have shown very limited colony forming potential in our previous studies with CFU-TOT counts of 0.1 and 0.6 per 1000 cells, respectively.

CFU-GM colonies (mean 53.2%) and CFU-GEMM colonies (mean 32.6%) were the most common colony types formed by of CB-derived HSCs. The proportion of different colony types for CD34+, CD133+, Lin- and MNCs is shown in Table 1. BFU-Es represented a mean of 12.9% of the colony content of HSCs. However, the Lin- cell fraction formed a surprisingly large number of BFU-Es (27.3%), probably due to the inefficient removal of erythroid progenitors during the depletion procedure. Very little CFU-E colonies were observed (mean 1.3%), MNC population being the most efficient in forming them (5.5%). The high proportion of BFU-E and CFU-E colonies formed by the MNC population reflects the unusual sedimentation of erythroid progenitors in Ficoll-Paque density gradient. The results show that all the selected HSC populations have substantial clonogenic potential and are highly non-committed. Taken together, the data support the use of traditionally used markers to separate HSC populations until more specific markers are found.

**Table 1:** Frequency of different types of CFU colonies within CD34+, CD133+, Lin- and MNC populations.

**Cell type**	**CFU-GM**	**CFU-GEMM**	**BFU-E**	**CFU-E**
CD34+	58.2%	33.2%	7.2%	1.4%
CD133+	57.4%	38.3%	4.3%	0.0%
Lin-	43.8%	26.3%	27.3%	2.6%
MNC	37.8%	41.3%	15.4%	5.5%

## Conclusion

Immunomagnetic cell sorting enables fast and gentle separation of HSCs. However, the previously reported protocols are not optimal for CB and result in unsatisfactory purity and yield, indicating a need for optimization of the procedures. With the modified protocols presented here, over 90% pure HSC fractions can be reproducibly obtained. This is essential for the use of specific hematopoietic progenitor cell types in research and therapeutic applications.

The single most important factor influencing engraftment in HSC transplantation appears to be the nucleated cell content. Even though the cell content is limited in CB and there is no possibility to obtain an additional graft from the same donor, the increased engraftment potential of CB-derived HSCs makes them an appealing alternative for HSCs from PB or BM. It remains to be seen whether the total nucleated cell content or a population of highly pure and specific hematopoietic progenitor cells will prove to be more important for graft potency.

## Methods

### CB units

Umbilical CB was obtained from informed and consenting donors at the Helsinki University Central Hospital, Department of Obstetrics and Gynaecology. Permit to collect and use donated stem cells has been obtained from the ethics board of the Helsinki University Central Hospital (550/E8/02) and the ethics board of the Finnish Red Cross Blood Service (40/02).

The umbilical cord was clamped according to standard hospital procedure and CB collections were performed *ex utero*. CB was collected into a sterile collection bag (Cord Blood Collection system, Medsep Corporation, Covina, USA) containing 25 ml of Citrate Phosphate Dextrose solution. The collection volume varied between 45–105 ml. Some CB units were volume reduced and cryopreserved in a BioArchive system at the Finnish Red Cross Blood Service, Cord Blood Bank as previously described [16,19] and some units were processed freshly within hours from collection. Altogether, 8 cryopreserved and 12 fresh CB units were used to optimize the protocols. In addition, 10 cryopreserved and 9 fresh CB units were used to test the optimized protocols.

### Handling of CB units

Cryopreserved CB unit was taken from the BioArchive system and kept for 2 minutes in the gas phase of liquid nitrogen, 3–5 minutes at room temperature and then in 37°C water bath until completely thawed. CB was transferred from the freezing bag into 50 ml tubes containing 10 ml of freezing solution: 2.5% albumin (Finnish Red Cross, Blood Service, Helsinki, Finland), 50% NaCl (Baxter Healthcare) and 50% Gentran40 (Baxter Healthcare). Cells were pelleted by centrifugation at 600 × g for 10 minutes and the supernatant was discarded. When cell aggregation occurred, the cell pellet was resuspended in 200 μl of 1 mg/ml DNaseI (Sigma-Aldrich, Steinheim, Germany). Cells were then suspended carefully in 100 ml of phosphate-buffered saline (PBS, pH 7.4) supplemented with 0.6% ACD/A. Fresh CB was diluted 1:4 with PBS supplemented with 2 mM EDTA, to reduce the size and number of cell aggregates and to give better lymphocyte yield in density gradient centrifugation.

MNCs were isolated by density gradient using 15 ml of Ficoll-Paque reagent (Amersham Biociences, Piscataway, USA) and 35 ml of diluted CB. The two-phase system was centrifuged at 400 × g for 40 minutes. MNCs, collected from the interface of the two phases, were washed twice with PBS. MNC counting was performed by automatic cell counter Sysmex K-1000 (Sysmex Corporation, Kobe, Japan).

### Separation of CD34+/CD133+ cells

CD34+ and CD133+ cells were enriched through positive selection using MiniMACS or MidiMACS separation systems (Miltenyi Biotec). For the labeling of CD34+ cells, Direct CD34 Progenitor Cell Isolation Kit was used, whereas CD133+ cells were labeled using the CD133 Cell Isolation Kit. 100 μl of FcR Blocking Reagent, to inhibit unspecific or Fc-receptor mediated binding, and 100 μl of CD34/CD133 MicroBeads for magnetic labeling of cells were added per 10^8 ^cells, as per manufacturer's recommendations.

MS or LS MACS affinity columns were used depending on the number of MNCs. MS column was used for up to 2 × 10^8 ^MNCs, and LS column was used for 2 × 10^8 ^to 2 × 10^9 ^of MNCs. Labeled cell suspension was subjected to immunomagnetic separation where magnetically labeled cells retain in the column while the unlabeled cells pass through the column. After several washes, the column was removed from the magnet and the retained CD34+ or CD133+ cells were eluted with 1–5 ml of PBS supplemented with 0.5% bovine serum albumin and 2 mM EDTA. CD34+ and CD133+ cells were subjected to one or two rounds of separation and their negative counterparts were collected for control purposes. In the two-column system, an additional labeling step between the column separations was tested, using 25 μl of both FcR Blocking Reagent and MicroBeads. The optimum purity and yield was obtained when using the additional labeling step in connection with the two-column system.

### Separation of Lin- cells

To enrich progenitor cells, lineage committed cells were depleted. MNCs (8 × 10^7^/ml) were labeled with Progenitor Enrichment Cocktail containing antibodies against CD2, CD3, CD14, CD16, CD19, CD24, CD56, CD66b and Glycophorin A (StemCell Technologies, Vancouver, Canada) at room temperature for 15 minutes. Subsequently, the cell suspension was incubated with magnetic iron particles at room temperature for 15 minutes. Varying concentrations of StemSep Progenitor Enrichment Cocktail (100 μl/ml, 75 μl/ml, and 50 μl/ml) and magnetic iron particles (60 μl/m, 45 μl/ml, and 30 μl/ml) were tested. Cell suspension was loaded into MACS LD column (Miltenyi Biotec) and unlabeled cells passing through the column were collected (Lin- fraction). The column was then washed twice with 1 ml of buffer and the remaining Lin+ cells were collected for control purposes. The conditions resulting in optimal separation of Lin- cells were 100 μl of Progenitor Enrichment Cocktail and 60 μl of magnetic iron particles per 8 × 10^7^/ml MNCs, as recommended by the manufacturer.

### Purity

To determine the purity of CD34+/- and CD133+/- cell fractions, 1 × 10^5 ^cells were labeled with fluorescein isothiocyanate (FITC)-conjugated anti-CD45 (clone 2D1, Becton Dickinson, Franklin Lakes, USA) and phycoerythrin (PE)-conjugated anti-CD34 (clone 345802 Becton Dickinson) or PE-conjugated anti-CD133 (clone 293C3, Miltenyi Biotec) monoclonal antibodies at 4°C for 15 minutes. Lin-/+ cells were labeled with PE-conjugated anti-mouse immunoglobulin specific polyclonal antibody (BD Biosciences, San Jose, USA). Platelets were detected with PE-conjugated anti-CD41a monoclonal antibody (BD Biosciences, San Jose, USA). Isotype-identical monoclonal antibodies served as controls. Labeled cells were analyzed using Becton Dickinson FACSCalibur™ with a 488 nm blue argon laser. Fluorescence was measured using 530/30 nm (FITC) and 585/42 nm (PE) bandpass filters. Data were analyzed using the ProCOUNT™ software (BD Biosciences) or Windows Multiple Document Interface for Flow Cytometry, WinMDI version 2.8 [23]. CD34+, CD133+ and Lin- cell populations were defined by first gating on forward and side scatter properties excluding platelets and debris. Subsequent gates were set to exclude >99% of control cells labeled with isotype-specific antibody.

### Colony forming unit assay

MNCs (1 × 10^5^) and enriched HSCs (2 × 10^3^) suspended in 300 μl Iscove's Modified Dulbecco's Medium supplemented with 2% fetal bovine serum (Gibco/Invitrogen, Paisley, United Kingdom) were mixed vigorously with 3 ml of MethoCult GF H4434 containing recombinant cytokines and erythropoietin (StemCell Technologies). The cells in MethoCult medium were plated in duplicate into sterile 35 mm petri dishes, and colonies were scored according to their morphological characteristics by light microscopy after a 14-day culture. Cell culture and colony scoring were performed according to accredited methodology (Finnish Cord Blood Bank, Finnish Red Cross Blood Service, Helsinki, Finland) following international standards [24,25]. The CFU assay was performed in triplicate for each cell type (CD34+, CD133+, Lin- and MNC).

### Statistical analysis

For statistical analysis of total MNC counts between cryopreserved and fresh CB samples, the non-parametric Mann-Whitney test was used, as the MNC counts were not normally distributed. The two groups, cryopreserved and fresh CB, were considered independent as they have been produced using different protocols. The recovery of CD34+, CD133+ and Lin- cells between cryopreserved and fresh CB was compared in independent samples by the t test and equality of variances was checked by Levene's test. When the assumption of equality was not satisfied, as with Lin- cells, the t test for non-equal variances was used to compensate the lack of homoscedasticity. P values less than 0.05 were considered statistically significant. The analyses were performed by Microsoft Excel and SPSS 12.0.1.

## Authors' contributions

TK and TJ contributed equally to the work and participated in the design of the study, optimized the experimental procedures, performed the mononuclear cell fractionation, HSC selections and flow cytometric analyses, and drafted the manuscript. SM performed the CFU assays and participated in the interpretation of data. JL participated in the design and coordination of the study, and assisted in drafting the manuscript. All authors read and approved the final manuscript.
